# ‘No to unnecessary caesarean sections’: Evaluation of a mass-media campaign on women’s knowledge, attitude and intention for mode of delivery

**DOI:** 10.1371/journal.pone.0235688

**Published:** 2020-08-11

**Authors:** Maedeh Majlesi, Ali Montazeri, Fatemeh Rakhshani, Elmira Nouri-Khashe-Heiran, Nahid Akbari

**Affiliations:** 1 Department of Midwifery and Reproductive Health, School of Nursing and Midwifery, Iran University of Medical Sciences, Tehran, Iran; 2 Health Metrics Research Center, Iranian Institute for Health Sciences Research, ACECR, Tehran, Iran; 3 Faculty of Humanity Sciences, University of Science &Culture, ACECR, Tehran, Iran; 4 Department of Public Health, School of Public Health, Shahid Beheshti University of Medical Sciences, Tehran, Iran; Medical Research Council, SOUTH AFRICA

## Abstract

**Introduction:**

Improvement of women’s knowledge and attitude toward vaginal birth is recognized as an important strategy to control caesarean sections (CS) on maternal request. This study aimed to evaluate the effectiveness of a mass-media campaign in improving knowledge, attitude and intention of women for vaginal birth.

**Methods:**

This was a population-based study carried out in Tehran, Iran. A national ‘No to unnecessary caesarean sections’ campaign was launched in April 2016 and was televised for ten days. A random sample of pregnant women from all defined geographical areas of Tehran were recruited and assessed for knowledge about the benefits of vaginal birth and the risk of CS, attitude and intention toward mode of delivery at two points in time: before and after the campaign. A comparison was made to evaluate outcome measures among those who had seen the campaign and those who had not.

**Results:**

In all, 37 public and private maternity care centers were selected randomly and 702 eligible pregnant women attending these centers were entered in the study. Pre- and post-intervention data for 466 women were available for analysis. Of these, 194 women indicated that they had seen the campaign and the remaining 272 women said that they had not. A comparison of the outcome measures between the two study groups showed that there were significant differences between those who had seen the campaign and those who had not. Those who had seen the campaign reported increased knowledge, had a more positive attitude and indicated increased behavioral intention toward vaginal birth.

**Conclusions:**

In general, the findings indicated that the mass-media campaign improved pregnant women’s knowledge, attitude and intention towards vaginal birth. However, the long-term effects of such campaigns need further investigation.

## Introduction

Caesarean section (CS) is a life-saving procedure in emergency obstetric care. However, high rates of CS have been linked to a number of potential risks and adverse consequences for maternal and child health [1, 2]. Recent international reports have shown that there is a rising trend in middle and high-income countries. The World Health Organization (WHO) recently reported that 48 countries had a CS rate of from 19.1% to 27% and that it was higher than 27% in 53 countries. It is argued that an increase in CS deliveries could create extra pressure on limited resources for health care systems in developing countries [3, 4].

Studies on current rates of caesarean section worldwide reported that 18.6% of all births occur by CS, ranging from 6% to 27.2% in the least and most developed regions, respectively. Latin America and the Caribbean region have the highest CS rates (40.5%), followed by Northern America (32.3%), Oceania (31.1%), Asia (19.2%) and Africa (7.3%) [5, 6]. In the European region, this rate was reported to be from 19% to 33% [7]. The highest rate of CS delivery occurs in countries such as China (64%), Dominican Republic (56.4%), Brazil (55.6%), Egypt (51.8%) and Colombia (43%) [3, 8]

Similarly, recent reports from Iran show that there is an upward trend in the CS rate from 21% in 2006 to 54% in 2016. The trend illustrates a significant annual rise in primary versus repeated CS rates during these years. A meta-analysis from Iran showed that the CS rate was from 51% to 90% in private hospitals, 48% in public hospitals and that 22% of caesarean sections were performed without clinical indications or maternal request [9].

Several factors could influence women’s choice of a caesarean section including health care-, individual-, and cultural-related factors [6, 10, 11]. Health care-related factors usually include policies and laws, and a dominant physician-patient relationship [12, 13]. Individual and cultural-related factors include fear of labor pain, poor knowledge, women’s socioeconomic status, previous experiences, negative attitudes and beliefs towards vaginal birth, misconceptions, social norms, and pressure from certain people, friends and family, rumors, and false complications attributed to vaginal birth [4, 14–16]. Thus, improving knowledge and modifying women’s attitudes toward vaginal birth are recognized as important strategies for controlling unnecessary CS and maternal requests to have a CS delivery [17, 18]. In doing so, different approaches were implemented including the use of mass-media campaigns. Although not fully successful, the findings from studies on mass-media campaigns have shown that these campaigns could improve knowledge, modify attitudes and change health-risk behaviors [19, 20]. For instance, several studies have shown that mass-media campaigns could produce positive changes in reproductive health-related behaviors such as cervical cancer screening or sex-related behaviors [21–28]. Therefore, we aimed to design and implement a mass-media campaign to assess the impact of such campaigns on women’s delivery intentions, hoping that the campaign would encourage women to choose vaginal birth.

## Methods

### Design

This was a population-based study using a simple before/after design to evaluate a mass-media campaign on knowledge, attitude and intention for mode of delivery among pregnant women living in Tehran, Iran. The campaign was launched in the year 2016 (from April 11 to 20) following a new childbirth policy in Iran aimed at encouraging childbearing and controlling the rising trend in caesarean sections. The campaign was intended to persuade pregnant women to choose vaginal birth instead of an unnecessary caesarean section. The evaluation included a pre-assessment (10 days before the study commenced) and post-assessment (a month after the campaign ended) of the effectiveness of the campaign.

### The campaign

The campaign consisted of a 100-second televised film clip on mode of delivery, named ‘No to unnecessary caesarean sections.’ The content of the clip was developed and revised in five sessions by an expert panel including two advertising professionals, two academic obstetricians, three academic midwives and two experts in health education. The panel completed an evaluation checklist on the content, the central theme, the lighting, visual aspects, professional approach, understandability, the quality and the captions. These evaluations were then finalized and the clip was pilot tested with 50 women attending a health center for prenatal care. In general, they found the clip acceptable and thus ready to be aired. The clip was telecasted on four national channels (out of eight existing channels) and in a study setting on a local TV channel for ten consecutive days targeting women of reproductive age. It was broadcasted eight times per day (three times in the morning, three times in the afternoon and two times in the evening). The clip was produced in the national language (Persian) and it was exclusively a TV campaign created by a private company. The Iran Ministry of Health approved the campaign and materials and its production was jointly sponsored by the Islamic Republic of Iran Broadcasting (IRIB) and the Iran University of Medical Sciences. A detailed description of the clip is presented in Box 1.

**Box 1. Description of the campaign**

**Fig 1:**
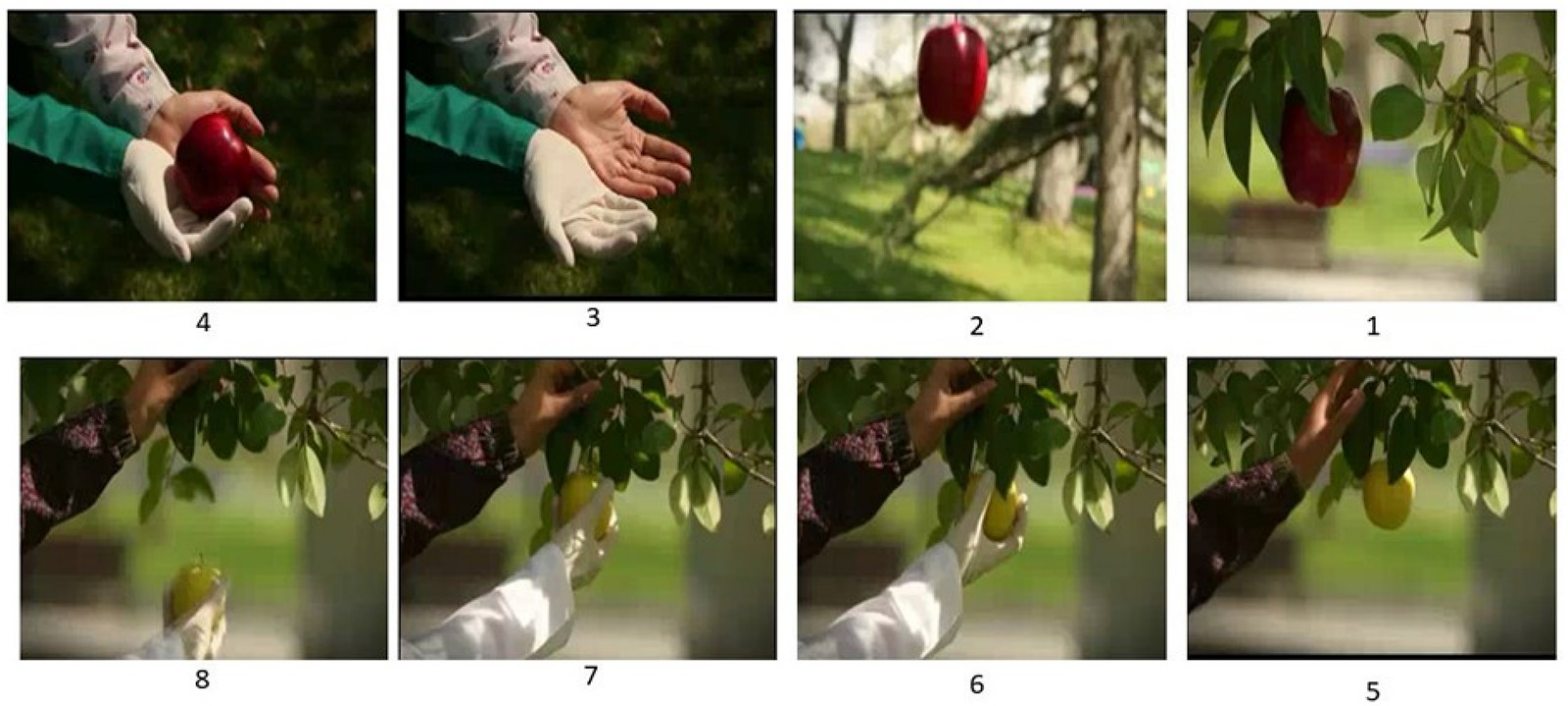
Pictures of the camaign.

The clip starts with a long shot of pleasant nature with the sound of birds (symbol of nature and human creation). After a few seconds, a garden with fruit trees is shown. The camera focuses on one of the apple trees (symbol of health). A ripe red apple with a thin stalk gradually separates from the tree branch (symbol of the onset of labor) and slowly falls with an eye-catching slow rotation (symbol of the rotation of the fetus in the mother’s pelvis) onto two hands—a mother’s hand (symbol of trust) and an unidentified woman’s hand (symbol of the midwife). At the same time, audiences hear a baby’s vigorous cry (symbol of a healthy baby). The clip goes on to show a branch of the tree on which hangs an immature green apple (symbol of incomplete growth and development of the fetus) being steadied by a mother’s hand (symbol of the maternal request for caesarean section), and then the hand of a woman plucking the apple from the branch (symbol of the medicalization of the natural process of birth). Then the clip shows the caesarean birth of a baby with a weepy and weak voice. The film ends with a view in which the narrator says: ‘Guarantee the health of your babies in the future by giving birth naturally.’ (See Fig 1, the clip can also be viewed at the following link: https://www.aparat.com/v/EJc2l).

### Settings and sample

**Fig 2:**
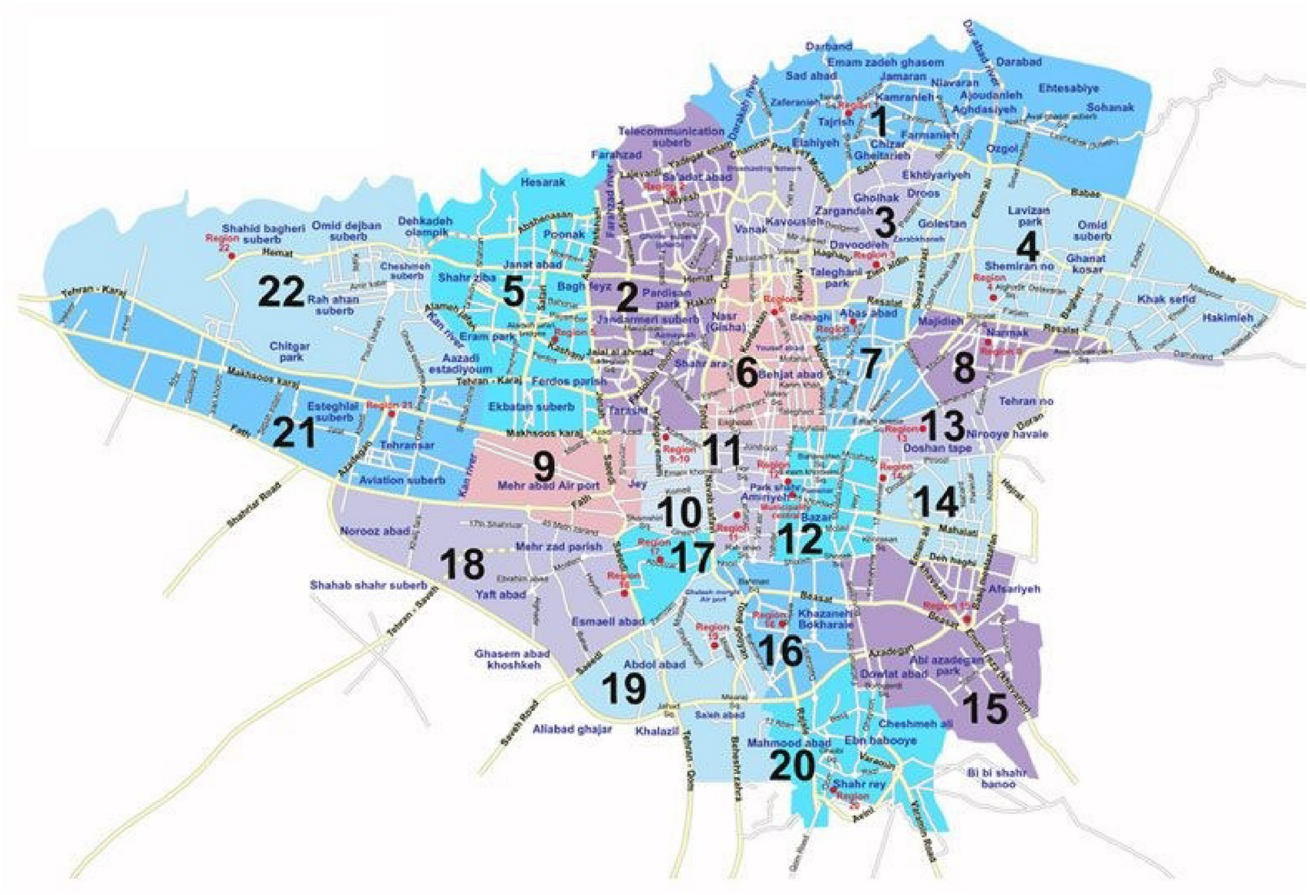
The map of Tehran (source: Municipality of Tehran, in public domain).

The study was conducted from April 10 to June 20, 2016 in Tehran, Iran. We used multi-stage cluster sampling. The study population was selected randomly from 22 municipal districts of Tehran tapping into five regions, namely: North, West, South, East and Center (Fig 2). Six districts including districts 3, 9, 11, 15, 17 and 21 were randomly selected for this study. Then, a list of all public and private centers was prepared. Finally, 37 public and 48 private centers were randomly selected from the list. Finally, convenient samples (a non-probability sample of women based on convenient accessibility) were recruited from each sampling unit. As such, we enrolled samples of pregnant women, regardless of their trimester, who attended the clinics on the day we started data collection and who agreed to participate in the study and provided written consent. Women with any history of CS, living outside Tehran, or with poor literacy and who had difficulty in understanding and answering the questionnaire were excluded.

### Instrument

A self-administered questionnaire was used. The first draft of the questionnaire was developed based on a review of relevant studies [29–32]. The questionnaire included three sections: items on demographic characteristics (age, level of education, income, occupation); reproductive characteristics (gravity; parity; previous experiences of birth delivery; gestational week; type of maternity care; source of information such as books, newspapers, radio, television, web and obstetricians/midwives/others); assessment of knowledge about the benefits of vaginal birth and the risks of caesarean section (8 items), attitudes toward vaginal birth (10 items) and attitudes toward caesarean section (6 items). Intention for mode of delivery was examined by a ‘yes/no’ format question. A panel of ten experts (three health education experts, six reproductive health experts and one epidemiologist) approved the final draft of the questionnaire. We used their comments to revise and modify the questionnaire. The expert panel was also used to make judgments about interpretation of the scores. The scoring procedure is described in Box 2.

**Box 2. Scoring procedure**

- **Knowledge**: The following scores were assigned to the response categories:
  No = 1, Do not know = 2 and Yes = 3.
  *Interpretation of the scores*: Respondents who scored between 8 and 12 were considered as not being aware of the benefits of vaginal birth and the risks of a caesarean section, a score between 13 and 18 as partially aware, and a score between 19 and 24 as substantially aware.
- **Attitude toward normal delivery**: The responses categories scored as follows:
  1 = Strongly disagree, 2 = Disagree, 3 = Neither agree nor disagree, 4 = Agree, and 5 = Strongly agree.
  *Interpretation of the scores*: A score between 10 and 22 was considered as a negative attitude, a score between 23 and 36 as a neutral attitude, and a score between 37 and 50 as a positive attitude towards vaginal birth.
- **Attitude toward caesarean section**: The response categories scored as follows:
  1 = Strongly disagree, 2 = Disagree, 3 = Neither agree nor disagree, 4 = Agree, and 5 = Strongly agree.
  *Interpretation of the scores*: A score ranging from 6 to 14 was considered a negative attitude, a score between 15 to 23 as a neutral attitude, and a score between 24 to 30 as a postive attitude towards a caesarean section.
- **Intention**: This was indicated as:
  ‘Intend to have a vaginal birth’ and ‘Intend to have a cesarean section.’
  The cumulative frequency and percentages for each option were reported.

### Statistical analysis

The data were analyzed for respondents for whom both pre- and post-campaign data were available using the SPSS software version 23. Chi-squared test was used to compare categorical data and the t-test was used to compare quantitative data. Statistical significance was set at the 0.05 level.

### Ethics

The Ethics Committee of the Iran University of Medical Sciences, Tehran, Iran approved the study (IR.IUMS.REC.1394. 92113732). All participants signed an informed consent form prior to any involvement. However, since a number of respondents under the age of 18 enrolled in the study, it should be noticed that according to the law the formal age for marriage in Iran for females is 13, and those who marry even under the age of 18 have the right to sign any legal documents including consent forms.

## Results

**Fig 3:**
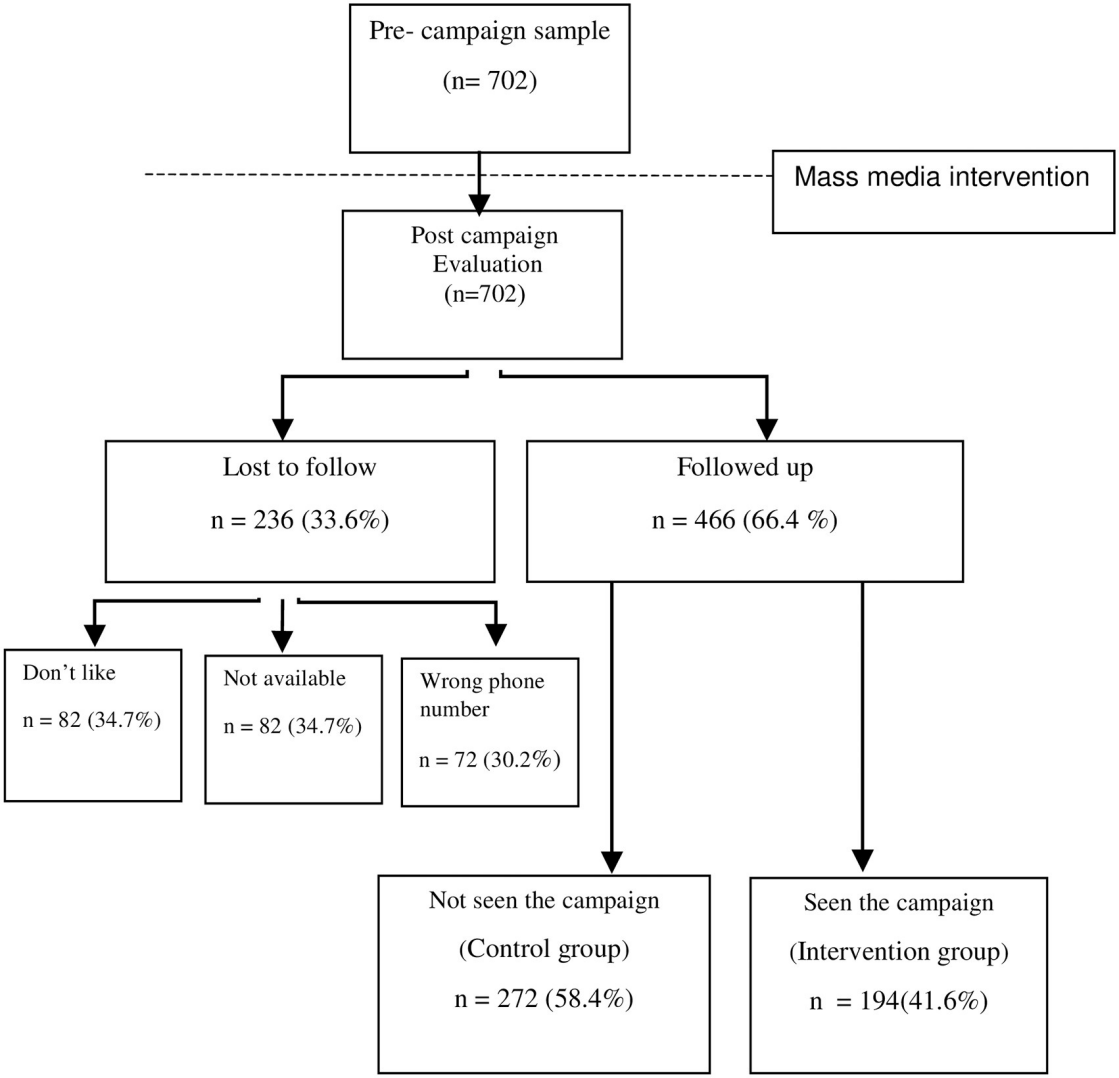
The study flowchart.

In all, 702 pregnant women were approached and completed and returned the questionnaire at baseline. However, at the follow-up assessment, only 466 women participated in the study (66.4% response rate) and the remaining 236 women were lost due to several reasons. Fig 3 shows the study flowchart. Thus, the analysis was limited to those for whom both baseline and follow-up data were available. The mean age of respondents was 28.69 ± 5.15 years. Overall, 49.9% of participants attended public hospitals and 50.1% attended private hospitals. A majority (50.7%) of women had a postgraduate education. In addition, 77.6% of expectant mothers were housewives. In general, there were no significant differences between the original sample and those who took part in the study. However, the missing sample seemed to be better off than those who took part in the study. In addition, the only difference between those who did respond and those who did not was that non-responders were more likely to have private maternity care. The characteristics of the study samples are shown in Table 1.

**Table 1:** Demographic characteristics of the study samples.

	Baseline (n = 702)	Followed up (n = 466)	Lost to follow (n = 236)		
	No. (%)	No. (%)	No. (%)	P-value*	P-value**
**Age**				0.771	0.648
15–20	46 (6.6)	32 (6.9)	14 (5.9)		
21–25	145 (20.7)	93 (20)	52 (22)		
26–30	255 (36.6)	173 (37.1)	82 (34.7)		
31–35	197 (28.1)	131 (28.1)	66 (28)		
>36	59 (8.4)	37 (8)	20 (9.3)		
**Educational level**				0.088	0.410
Illiterate/primary	110 (15.7)	79 (16.9)	31 (13.1)		
Secondary education	243(34.6)	165 (35.4)	78 (33.1)		
Higher education	349 (49.7)	222 (47.6)	127 (53.8)		
**Occupation**				0.538	**0.022**
Housewife	545(77.6)	365 (78.3)	180 (76.3)		
Employed	157(22.3)	101 (21.7)	56 (23.7)		
**Income level**				0.191	0.068
Poor	303(43.2)	215 (46.1)	88 (37.3)		
Fair	338(48.1)	207(44.4)	131 (55.5)		
Good	61(8.7)	44 (9.4)	17 (7.2)		
**Source of information**				0.690	0.880
TV& radio	136 (19.2)	90 (19.3)	46(19.5)		
Book & press	83 (11.9)	59 (12.7)	24 (10.2)		
Internet & Social media	228 (32.5)	138 (29.6)	90 (38.1)		
Family & friend	38 (5.5)	34 (7.3)	4 (1.7)		
Doctor & midwife	217 (30.9)	145 (31.1)	72(30.5)		
**Types of maternity care centers**				0.115	**0.005**
Public	350(49.9)	250 (53.6)	100 (42.4)		
Private	352(50.1)	216 (46.4)	136 (57.6)		
**Trimester**				0.489	0.767
1^st^	158 (22.5)	108 (23.2)	50 (21.2)		
2^nd^	267 (38)	178 (38.2)	89 (37.7)		
3^rd^	277 (39.5)	180 (38.6)	97 (41.1)		
**Parity**				0.165	0.155
One	500 (71.2)	324 (69.5)	176 (74.6)		
Two	142 (20.2)	99 (21.2)	43 (18.2)		
Three and more	60 (8.5)	43 (9.3)	17 (7.2)		

* Significant level between 702 & 466.

** Significant level between 466 & 236.

As mentioned, 466 pregnant women were followed up after the mass-media campaign. Of these, 194 respondents reported that they had seen the campaign (41.6%) and 272 respondents said that they had not (58.4%). The majority of women who had seen the campaign indicated that the campaign was appealing and they liked it (85%). A comparison of knowledge, attitude toward vaginal birth and caesarean section between those who had seen the campaign and those who had not is presented in Table 2. At baseline, there were no significant differences in knowledge and attitude between those pregnant women who had seen the campaign and those who had not. However, at the follow-up assessment, significant differences were observed for knowledge (P = 0.018) and attitude toward vaginal birth (P = 0.030), but not for attitude toward caesarean section (P = 0.415). In addition, those who had seen the campaign showed significant improvements in knowledge and attitude toward vaginal birth, while the attitudes of those who had not seen the campaign remained unchanged.

**Table 2:** Knowledge and attitude of participants regarding mode of delivery (n = 466)*.

	Seen (n = 194)	Not seen (n = 272)	
	Mean (SD)	Mean (SD)	P**
**Pre-campaign score**			
Knowledge	17.08 (4.0)	17.03 (3.96)	0.862
Attitude toward vaginal birth	39.13 (6.71)	38.01 (7.23)	0.091
Attitude toward CS	14.90 (3.85)	15.20 (4.01)	0.664
**Post campaign score**			
Knowledge	18.08 (3.45)	17.68 (3.04)	0.018
Attitude toward vaginal birth	40.07 (5.21)	39.01 (5.22)	0.030
Attitude toward CS	14.44 (2.16)	15.35 (2.08)	0.415

* Higher scores indicate better knowledge and positive attitudes toward a given mode of delivery.

** Derived from independent samples t-test.

Comparing intentions toward vaginal birth and caesarean section between those who had seen the campaign and those who had not also showed that after the campaign there was a significant increase in the proportion of women who indicated that they intend to seek vaginal birth (P = 0.004, chi-squared). The detailed results are shown in Table 3.

**Table 3:** Behavioral intention of participants regarding mode of delivery (n = 466).

	Pre-campaign intention	Post campaign intention	P-value*
	No. (%)	No. (%)	
**Seen** (n = 194)			0.004
Vaginal birth intention	118 (60.8)	146 (75.3)	
CS intention	76 (39.2)	48 (24.7)	
**Not seen** (n = 272)			0.172
Vaginal birth intention	186 (68.4)	201(73.9)	
CS intention	86 (31.6)	71 (26.1)	

* Derived from Chi-square test.

## Discussion

We found that the mass-media campaign was effective in improving women’s knowledge, attitude and intention to seek vaginal birth. Similar findings were reported for topics related to reproductive health. For instance, studies have shown that media campaigns have successfully improved knowledge and changed attitudes and reproductive health behaviors [10, 28, 33].

The findings from the current study indicated that overall 58.4% of women said that they did not see the campaign. This seems to be a disappointing rate and needs to be carefully investigated. One way to achieve this might be to ask respondents a simple question to indicate why they did not see the campaign. Unfortunately, the current study did not investigate this, but we speculate that these women only see Iranian TV occasionally and they use social media more frequently. Thus, it is useful for future programs to consider those women who usually are not interested in formal communication channels and prefer to use their own social media channels. Furthermore, the impact of a mass-media campaign on a population that includes a relatively high proportion of illiterate or poorly educated women (16.9% of those who provided data) is an important issue that should be noticed. Another useful suggestion for improving exposure might be to ask women who did see the campaign to state how that occurred.

The role of providers and women is important in making decisions about mode of delivery. Thus, to reduce the number of unnecessary CSs, comprehensive interventions should be implemented, both to improve women’s preferences and change providers’ behaviors. At present, several national regulations have been implemented to encourage obstetricians and midwives to perform vaginal births. However, recent studies have shown that factors such as poor supportive system, staff shortages and poor attitudes of obstetricians toward vaginal birth have influenced decisions on mode of delivery by pregnant women [33–39].

After the campaign, women’s preferences and intentions for CS did not change significantly. It seems that strong perceived social pressure regarding ‘CS delivery as a luxurious and safe delivery’ has strengthened women’s attitudes and their intentions to have a CS delivery [35]. Perhaps in countries with a high rate of CS such as Iran, the potential risks of elected CS delivery have been overshadowed by obstetrics and social pressure. We believe that investment in longer mass-media campaigns could lead to a modification of attitudes towards CS among women who feel strong social pressures. Furthermore, exposure to fear-based messages [36] about the risks associated with elected CS delivery, especially the risks for their babies, might be more effective in motivating them to choose vaginal birth.

This study had some strengths and limitations. The strengths included the fact that it was a population-based study. Secondly, the study benefited from an expert panel with different specialties in developing the campaign and thus making the campaign as acceptable as possible to all disciplines and audiences. However, the limitations included loss of a large number of women from the study sample at the follow-up assessment. In addition, women in this study, by virtue of being in the study, were probably more aware of the campaign, and so may have given the campaign more attention than other pregnant women who were not subject to the baseline questionnaire. Moreover, when asking women about their intention toward mode of delivery, the respondents could only select ‘yes’ or ‘no’ from the response categories and we overlooked inserting an option for those who were ‘not sure’ or would like to respond ‘don’t know.’ Finally we did not collect information on the frequency of exposure to the campaign. This might have influenced the results. We recommend that in future investigations, questions are asked about the frequency of exposure.

## Conclusion

Overall, the findings suggest that media campaigns play a significant role in supporting programs for vaginal birth on a large scale. In fact media campaigns could improve knowledge, attitudes and intentions towards vaginal birth. However, longitudinal investigations will be required to uncover the real decisions for vaginal birth following media campaigns.

## Supporting information

S1 Data Minimal set of the data. (SAV)
